# SAP-Dependent and -Independent Regulation of Innate T Cell Development Involving SLAMF Receptors

**DOI:** 10.3389/fimmu.2014.00186

**Published:** 2014-04-23

**Authors:** Jaime De Calisto, Ninghai Wang, Guoxing Wang, Burcu Yigit, Pablo Engel, Cox Terhorst

**Affiliations:** ^1^Division of Immunology, Beth Israel Deaconess Medical Center, Harvard Medical School, Boston, MA, USA; ^2^Immunology Unit, Department of Cell Biology, Immunology and Neurosciences, Medical School, University of Barcelona, Barcelona, Spain

**Keywords:** SLAM, SAP, innate-like lymphocytes, NKT, innate CD8^+^ T cells, T cell development, PLZF, Eomes

## Abstract

Signaling lymphocytic activation molecule (SLAM)-associated protein (SAP) plays an essential role in the immune system mediating the function of several members of the SLAM family (SLAMF) of receptors, whose expression is essential for T, NK, and B-cell responses. Additionally, the expression of SAP in double-positive thymocytes is mandatory for natural killer T (NKT) cells and, in mouse, for innate CD8^+^ T cell development. To date, only two members of the SLAMF of receptors, Slamf1 and Slamf6, have been shown to positively cooperate during NKT cell differentiation in mouse. However, it is less clear whether other members of this family may also participate in the development of these innate T cells. Here, we show that *Slamf[1* + *6]^−/−^* and *Slamf[1 *+ *5 *+ *6]^−/−^*B6 mice have ~70% reduction of NKT cells compared to wild-type B6 mice. Unexpectedly, the proportion of innate CD8^+^ T cells slightly increased in the *Slamf[1 *+ *5 *+ *6]^−/−^*, but not in the *Slamf[1 *+ *6]^−/−^* strain, suggesting that Slamf5 may function as a negative regulator of innate CD8^+^ T cell development. Accordingly, *Slamf5^−/−^* B6 mice showed an exclusive expansion of innate CD8^+^ T cells, but not NKT cells. Interestingly, the SAP-independent *Slamf7^−/−^* strain showed an expansion of both splenic innate CD8^+^ T cells and thymic NKT cells. On the other hand, and similar to what was recently shown in *Slamf3^−/−^* BALB/c mice, the proportions of thymic promyelocytic leukemia zinc finger (PLZF^hi^) NKT cells and innate CD8^+^ T cells significantly increased in the SAP-independent *Slamf8^−/−^* BALB/c strain. In summary, these results show that NKT and innate CD8^+^ T cell development can be regulated in a SAP-dependent and -independent fashion by SLAMF receptors, in which Slamf1, Slamf6, and Slamf8 affect development of NKT cells, and that Slamf5, Slamf7, and Slamf8 affect the development of innate CD8^+^ T cells.

## Introduction

During mainstream T lymphocyte development, some of the key factors that drive the transition from double-positive (DP) CD4^+^CD8^+^ precursors to single positive (SP) T cells involve antigen specificity and TCR strength (1–3). During this process, thymic epithelial cells interact with DP cells to determine the fate of immature thymocytes by orchestrating positive and negative selection (4–7). By contrast, homotypic DP–DP interactions drive development and selection of the so-called innate αβ T lymphocytes, which include natural killer T (NKT) and innate CD8^+^ T cells (8–10). During development in the thymus, as well as in the effector phase in the periphery, NKT and innate CD8^+^ T cells, which have a restricted TCR repertoire, interact with different non-classical MHC class I molecules (11–14). These cells are thought to be the *first responder* cells that can rapidly release various cytokines and control both viral and bacterial infections (15).

Signaling lymphocytic activation molecule (SLAM)-associated protein (SAP) (encoded by *Sh2d1a* in mouse) and several SLAM family (SLAMF) receptors provide DP thymocytes with positive signals that favor their maturation in the thymus (16–20). Cognate activation of NKT cells is restricted to CD1d–lipid complexes and is modulated by SAP and at least three members of the SLAMF of receptors (20–22). The homophilic interactions of Slamf1 and Slamf6 between DP thymocytes are particularly important for the development of the NKT cell lineage (20). Binding of SAP to the immunoreceptor tyrosine-based switch motifs (ITSMs) present in the cytoplasmic tail of several SLAMF receptors (23–25) promotes a unique interaction between the active configuration of the Src tyrosine kinase Fyn and the SLAMF receptor (26, 27), while at the same time blocking the recruitment of the protein phosphatases SHP-1 and SHP-2 (28–30), leading to efficient T cell activation and survival (31, 32). Recent evidence now demonstrates that Slamf3, another SAP-associated SLAMF receptor, acts as an inhibitory receptor for NKT and innate CD8^+^ T cell development (22). This suggests that differential SLAMF receptor expression can positively or negatively influence innate T cell development.

Non-conventional innate CD8^+^ T cells are also selected in the thymus from DP progenitors upon interaction with hematopoietic cells. Their TCR specificity is restricted to non-classical MHC class Ib molecules, including H2-M3 (histocompatibility 2, M region locus 3), Qa-1 (H2-T23), and MR1 (MHC class I related) (15). Like NKT cells, innate CD8^+^ T cells bear an activated phenotype (CD44^hi^CD122^+^) and promptly produce interferon-gamma (IFN-γ) upon activation. Moreover, positive selection of these innate CD8^+^ T cells in the thymus strictly depends on interleukin (IL)-15 (33–36). These lymphocytes have been most extensively described in *Rlk^−/−^Itk^−/−^* (resting lymphocyte kinase/iterleukin-2-inducible T cell kinase) and *Itk^−/−^* B6 mice, where these kinases are believed to set the threshold of TCR activation during lineage commitment. Hence, T cell clones with high MHC affinity will escape negative selection and acquire innate-like features (3, 15, 37–39). Notably, the expansion of these cells in *Itk^−/−^* mice, and in other deficient B6 mouse strains with a similar phenotype (10, 40) depends on a subset of thymic promyelocytic leukemia zinc finger (PLZF^hi^) NKT cells producing IL-4 (41–43). Importantly, this process also requires an intact SAP expression in the hematopoietic compartment (41). Sensing of IL-4 by developing innate CD8^+^ T cells upregulates one of the key transcription factors involved in the acquisition of the innate-like program by these cells, the T-box transcription factor, Eomesodermin (Eomes) (42–46). In turn, Eomes directs the expression of granzyme B, perforin, IFN-γ and, importantly, the expression of the IL-2/IL-15 receptor β chain, CD122, which conveys responsiveness to the cytokine IL-15 (44).

Mutations in the human *SH2D1A* gene lead to X-linked lymphoproliferative syndrome (XLP) (28). This rare inherited disorder is characterized by exaggerated T- and B-cell responses against Epstein–Barr virus (EBV), resulting in EBV-induced infectious mononucleosis, hypogammaglobulinemia, and a higher risk of developing various forms of lymphoma. Because patients with XLP lack NKT cells, the study of the regulation of their development and function by SLAMF receptors and SAP will shed light on the pathogenesis of this often-fatal disease. An important unanswered question in this field relates to how the coexpression of different arrays of SLAMF receptors favors the development of a particular innate T cell lineage. Therefore, in this study, we aimed to evaluate the relative contributions of three SAP-binding receptors, Slamf1, Slamf5, and Slamf6, and two SAP-independent receptors, Slamf7 and Slamf8, to the development of NKT and innate CD8^+^ T cells in the mouse.

## Materials and Methods

### Mice

*Slamf[1 *+ *6]^−/−^* and *Slamf[1 *+ *5 *+ *6]^−/−^*mice on a C57BL/6 (B6) are described by Wang et al. (manuscript submitted for publication). *Jα18^−/−^* mice on a B6 background, originally from Dr. Taniguchi (Riken, Yokohama, Japan), were provided by Dr. Exley (Beth Israel Deaconess Medical Center (BIDMC), Harvard Medical School, Boston, MA, USA). *Slamf7^−/−^* B6 mice (B6N.129S5-*Slamf7^tm1Lex^*/Mmucd, identification number 032574-UCD) were obtained from the Mutant Mouse Regional Resource Center (MMRRC), an NIH-funded strain repository, and was donated to the MMRRC by Genentech, Inc., *Sh2d1a^−/−^* mice on B6 and BALB/c backgrounds have been previously described (47). *Slamf8^−/−^* mice on a BALB/c background have been previously described (48). Age- and sex-matched controls on the B6 and BALB/c backgrounds were purchased from The Jackson Laboratory (Bar Harbor, ME, USA) or from Charles River Laboratories (Wilmington, MA, USA). All animals were housed in the Center for Life Science animal facility of the BIDMC. The experiments were performed according to the guidelines of the Institutional Animal Care and Use Committee at BIDMC.

### Generation of *Slamf5^−/−^* mice

A B6 background murine bacterial artificial chromosome (BAC) clone (BAC RP23-77A8) was used as the PCR template for cloning the 5′, 3′ arm and middle homologous fragments of the targeting vector. The PCR primers for the 6-kb 5′ targeting arm were designed from the upstream sequence of exon 1. The 2.1-kb middle arm that contains the promoter region plus exon 1, and the 4-kb 3′ arm intron 1 fragments were generated by PCR. The PCR products were cloned into a targeting vector containing a LoxP-FRT-Neo-FRT-LoxP cassette (Figure 2A). The resulting targeting construct was sequenced to confirm the correct sequences and orientation of the inserted PCR fragments. The targeting vector was then incorporated into Bruce 4 ES cells by electroporation. Southern blot hybridization was performed with a 5′ external probe to detect the homologous recombination event, and the positive clones were confirmed using a 3′ external probe. Four independent positive ES clones were microinjected into blastocysts from BALB/c mice and gave rise to germline transmission of the Slamf5 mutant DNA. The resulting mice were bred with FLP transgenic B6 mice. FLP-mediated recombination resulted in the deletion of the FRT-flanked *Neo* gene. Subsequently, *Neo*-free mice were bred with CreTg mice to generate *Slamf5^−/−^* mice (Figures 2A,B).

### Antibodies, tetramers, and flow cytometry

PLZF (Mags.21F), Eomes (Dan11mag), TCRβ (H57-597), CD3 (17A2), and CD122 (TM-b1) antibodies were purchased from eBioscience (San Diego, CA, USA). CD4 (RM4.5), CD8α (53–6.7), CD44 (IM7), IFN-γ (XMG1.2), Slamf3 (CD229), and Slamf5 (CD84) antibodies were purchased from BioLegend (San Diego, CA, USA). Slamf1 (CD150), Slamf2 (CD48), Slamf4 (CD244), and Slamf6 (CD352) antibodies were purchased from BD Pharmingen (San Diego, CA, USA). PBS-57-loaded CD1d tetramers were provided by the National Institutes of Health tetramer facility (Atlanta, GA, USA). Briefly, 2–5 × 10^6^ thymocytes or splenocytes were incubated with the relevant cocktail of antibodies for 30 min on ice, washed twice with PBS + 5% FCS + 5 mM EDTA, and acquired on a BD™ LSR II flow cytometer (BD Biosciences, San Jose, CA, USA). Dead cells were excluded by DAPI (Invitrogen, Carlsbad, CA, USA) or by using the Fixable Viability Dye eFluor^®^ 455UV reagent (eBioscience). Data analysis was performed using FlowJo software (TreeStar, Ashland, OR, USA). For specific details concerning the FACS gating strategy used in this manuscript, refer Figures S1 and S2 in Supplementary Material. To accurately identify the background levels of CD122, CD44, Eomes, and PLZF from the true positive population, the fluorescence minus one (FMO) approach, in which all dyes except for the one of interest are added to the samples, was used in all the FACS analysis (49–51).

### Intracellular staining

After surface staining (including the Fixable Viability Dye eFluor^®^ 455UV), cells were fixed in IC/Fixation buffer (eBioscience) and then incubated for 30 min on ice with anti-PLZF plus anti-Eomes antibodies in 1× permeabilization buffer (eBioscience). After several washes, the cells were acquired on a BD™ LSR II flow cytometer (BD Biosciences).

### *Ex vivo* cell activation and intracellular cytokine staining

Total thymocytes from wild-type (WT) or *Slamf8^−/−^* BALB/c mice were incubated overnight at 37°C in the presence of the Cell Stimulation Cocktail (plus protein transport inhibitors) reagent (eBioscience). After surface staining (including the Fixable Viability Dye eFluor^®^ 455UV), cells were fixed/permeabilized and incubated for 30 min with anti-IFN-γ antibody on ice. Samples were acquired on a BD™ LSR II flow cytometer. Data analysis was performed using FlowJo software.

### RNA isolation and real-time PCR (qPCR)

Total RNA was isolated using TRIzol^®^ LS reagent (Invitrogen) and precipitated with isopropanol. All RNA samples were tested in duplicates, at 50 ng/well, using the One-Step Real-Time RT-PCR Master Mix Reagent (Applied Biosystems^®^, Part Number 4309169). qPCR was performed and analyzed on the 7500 FAST Real-Time PCR System (Applied Biosystems^®^). *Slamf8* (Mm01293286_m1), *Eomes* (Mm01351985_m1), *Zbtb16* (Mm01176868_m1), *Irf4* (Mm00516431_m1), *IL-4* (Mm00445259_m1), and the Eukaryotic 18S ribosomal RNA Endogenous Control TaqMan^®^ probes were purchased from Life Technologies (Carlsbad, CA, USA). The relative gene-specific fold change, normalized to 18S rRNA, was calculated using the 2^−ΔΔct^ method and expressed relative to WT levels (WT = 1).

### Statistical analysis

Statistical significance was determined by unpaired *t*-test (two-tailed with equal SD) using Prism software (GraphPad, San Diego, CA, USA). The *p* value <0.05 was considered statistically significant.

## Results

### Differential expression of SLAMF receptors by mouse thymocyte subsets

Since both NKT and innate CD8^+^ T cells are selected from homotypic interactions between DP thymocytes, we thought that it was relevant to determine the specific SLAMF receptor expression pattern of these cells (Figure S1 in Supplementary Material). As previously reported (20, 22), we found that the homotypic SAP-dependent receptors Slamf1, Slamf3, Slamf5, and Slamf6 are moderately to highly expressed by DP TCRβ^low^ thymocytes. Although Slamf2 is highly expressed by these cells, its receptor, Slamf4, is virtually absent, suggesting that this heterophilic interaction does not play a role during homotypic DP-DP contacts (Figure S1D in Supplementary Material). On the other hand, CD44^hi^NK1.1^+^ mature thymic NKT cells showed less Slamf1 and Slamf6 expression than their DP progenitors, while Slamf2, Slamf3, Slamf4, and Slamf5 were upregulated (Figure S1D in Supplementary Material). Thymic innate CD8^+^ T cells maintained the levels of Slamf1, Slamf4, and Slamf6 expression compared to DP TCRβ^low^ thymocytes, but slightly upregulated Slamf2, Slamf3, and Slamf5 (Figure S1D in Supplementary Material). Once in the spleen, mature NKT cells expressed Slamf1, Slamf5, and Slamf6 at a higher level than in the thymus, but lacked Slamf4 expression. Slamf2 and Slamf3 levels did not change on these cells. With the exception of Slamf1 and Slamf6, which were downregulated, the other SLAMF receptors showed a similar level of expression in splenic innate CD8^+^ T cells compared to their thymic counterparts (Figure S1D in Supplementary Material). Together, these data suggest that the SAP-dependent receptors Slamf1, Slamf3, Slamf5, and Slamf6 may participate in early homotypic cell–cell interactions leading to NKT and/or innate CD8^+^ T cell differentiation.

### SAP-independent receptors Slamf7 and Slamf8 are highly expressed in thymic-resident dendritic cells

We used the Gene Skyline tool from the Immunological Genome Project’s website [Immgen.org; (52)] to determine *Slamf7*, *Slamf8*, and *Slamf9* mRNA expression levels by different thymic populations (Table 1). *Slamf7* mRNA was differentially expressed by mature NKT cells, dendritic cells (DC), and medullary epithelial cells, while *Slamf8* mRNA was only present in DC and thymic fibroblasts, but not in DP thymocytes. *Slamf9* mRNA was moderately expressed by DP thymocytes, DC, and medullary epithelial cells only. Furthermore, *Sh2d1a* mRNA (encoding SAP) was only detected in DP and mature NKT cells, while *Sh2d1b1* mRNA (encoding EAT-2A) was not expressed by any of the cell subsets analyzed. These data suggest that the SAP-independent SLAMF receptors, Slamf8 and Slamf9, may also modulate innate T cell differentiation in mice.

**Table 1:** **mRNA levels in SAP-independent SLAMF receptors and SLAMF adaptors expressed by different murine thymic populations**.

Cell subset	*Slamf7*	*Slamf8*	*Slamf9*	*Sh2d1a*	*Sh2d1b1*
DP thymocytes	−	−	+	+	−
Mature NKT	++	−	−	+	−
CD8^−^DC	+++	++	+	−	−
CD8^+^ DC	+++	++	+	−	−
Medullary epithelial cells	+	−	+	−	−
Thymic fibroblasts	−	+	−	−	−

*DP thymocyte: CD4^+^CD8^+^TCRβ^−/low^CD69^−^; mature NKT: TCRβ^+^PBS-57-CD1d-Tet^+^CD44^+^NK1.1^+^; DC: CD11c^+^MHC-II^+^CD11b^−^CD4^−^; medullary epithelial cells: CD45^-^EpCAM^+^Ly51^−^MHC-II^hi^; thymic fibroblast: CD45^-^PDGFRα^+^MTS15^+^. Symbols represent the relative normalized mRNA levels (arbitrary units) according to the following criteria: (−), <50; (+), 50–500; (++), 500–1000; (+++), >1000*.

### Slamf1 and Slamf6 control NKT but not innate CD8^+^ T cell development

Although disruption of *Slamf1* or *Slamf6* gene only marginally affects NKT cell development (20) (Table 2), the use of mixed-bone marrow chimeras or a lentivirus-based knockdown approach have suggested that these SLAMF receptors positively cooperate during NKT cell differentiation (20, 53). Therefore, to better understand the consequences of a combined deficiency of more than one SLAMF receptor during innate T cell development, we generated *Slamf[1 *+ *6]^−/−^* and *Slamf[1 *+ *5 *+ *6]^−/−^*B6 mice by two sequential homologous recombination-based gene disruptions (Wang et al., manuscript submitted for publication).

**Table 2:** **Effects of SAP and SLAMF receptor deficiencies on innate T cell development in the thymus of B6 mice**.

C57BL/6	*Sh2d1a^−/−^*	*F1^−/−^*	*F5^−/−^*	*F6^−/−^*	*F[1 *+ *6]^−/−^*	*F[1 *+ *5 *+ *6]^−/−^*	*F3^−/−^*	*F7^−/−^*
Mature NKT	↓ ↓ ↓	↓	±	↓	↓ ↓	↓ ↓	↑	↑
Innate CD8	↑	?	±	?	±	±	=	=
References	(17–19, 40, 41, 53–55)	(20, 56, 57)	This work	(20, 53, 56)	This work and (20)	This work	(22, 58)	This work

*↓, Reduction; ↑, expansion; ±, marginal/some effect; =, no effect; ?, no data available*.

**Figure 1:**
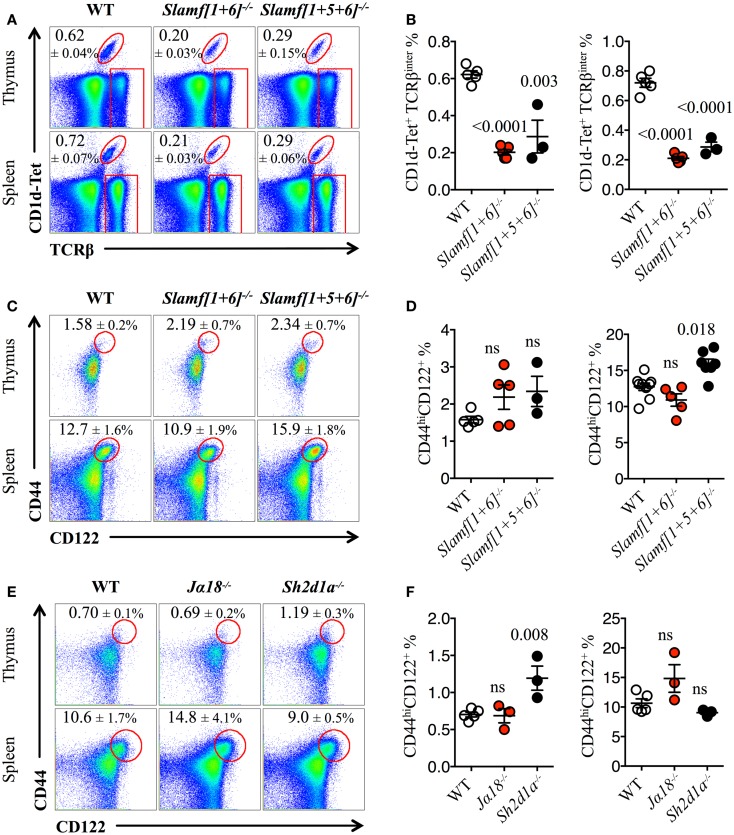
**Slamf1 and Slamf6 are required for NKT cell but not for innate CD8^+^ T cell development**. **(A)** Representative FACS plots showing PBS-57 CD1d-tetramer (CD1d-Tet) reactive NKT cells from thymus or spleen of WT, *Slamf[1 *+ *6]^−/−^* or *Slamf[1 *+ *5 *+ *6]^−/−^*B6 mice. FACS plots are gated on live singlets lymphocytes. **(B)** Percentage of CD1d-Tet^+^TCRβ^inter^ cells in thymus (*left*) or spleen (*right*) of WT, *Slamf[1 *+ *6]^−/−^* or *Slamf[1 *+ *5 *+ *6]^−/−^*B6 mice. **(C)** Representative FACS plots showing the expression of CD44 and CD122 on single positive CD8 T cells (CD8SP) from thymus or spleen of WT, *Slamf[1 *+ *6]^−/−^* or *Slamf[1 *+ *5 *+ *6]^−/−^*B6 mice. FACS plots are gated on live singlets lymphocytes TCRβ^hi^ CD8SP (*thymus*) or TCRβ^+^ CD8SP (*spleen*). **(D)** Percentage of CD44^hi^CD122^+^ CD8SP T cells in thymus (*left*) or spleen (*right*) of WT, *Slamf[1 *+ *6]^−/−^* or *Slamf[1 *+ *5 *+ *6]^−/−^*B6 mice. **(E)** Representative FACS plots showing the expression of CD44 and CD122 on CD8SP T cells from thymus or spleen of WT, *J*α*18^−/−^* or *Sh2d1a^−/−^* B6 mice. FACS plots are gated on live singlets lymphocytes TCRβ^hi^ CD8SP (*thymus*) or TCRβ^+^ CD8SP (*spleen*). **(F)** Percentage of CD44^hi^CD122^+^ CD8SP T cells in thymus (*left*) or spleen (*right*) of WT, *J*α*18^−/−^* or *Sh2d1a^−/−^* B6 mice. Numbers inside plots show the mean percentage ± SD of NKT cells **(A)**, or innate-like CD8^+^ T cells **(C, E)**. Red rectangles in **(A)** depict the lymphocyte gate in which innate CD8^+^ T cells were analyzed. Cumulative graphs show the results of two independent experiments, mean ± SEM. The *p* values were calculated by unpaired *t*-test between the WT group and the knockout group. A value of *p * < 0.05 was considered significant; ns, non-significant.

Both *Slamf[1 *+ *6]^−/−^* and *Slamf[1 *+ *5 *+ *6]^−/−^*B6 mice developed normally and had no gross phenotype as compared to their WT littermates (Wang et al., manuscript submitted for publication). Consistent with the outcomes of our previous bone marrow chimera experiments (20), NKT cell development was severely impaired as judged by a 70% reduced frequency and absolute cell counts (*data not shown*) of PBS-57 CD1d-tetramer (CD1d-Tet) reactive cells (NKT) both in the thymus and spleen of *Slamf[1 *+ *6]^−/−^* and *Slamf[1 *+ *5 *+ *6]^−/−^*B6 mice (Figures 1A,B). Importantly, these results further confirm the significance of Slamf1 and Slamf6 coexpression in DP thymic precursors for NKT cell development. Since the additional disruption of the *Slamf5* gene in the triple-knockout strain did not further reduce the NKT cell compartment (Figures 1A,B), we conclude that in the absence of Slamf1 and Slamf6, Slamf5 does not play a major role in NKT cell differentiation.

As NKT cells have been shown to support innate CD8^+^ T cell development (10, 43), we hypothesized that both *Slamf[1 *+ *6]^−/−^* and *Slamf[1 *+ *5 *+ *6]^−/−^*B6 mice would present a marked reduction in the proportions of innate CD8^+^T cells due to the dramatic loss of NKT cells (Figures 1A,B). Unexpectedly, we did not find a significant impairment of the innate CD8^+^ T cell pool (CD44^hi^CD122^+^) in the thymus of *Slamf[1 *+ *6]^−/−^* or *Slamf[1 *+ *5 *+ *6]^−/−^*B6 mice (Figures 1C,D). Instead, we found a slight increase of splenic innate-like CD8^+^ T cells in the absence of Slamf1, Slamf5, and Slamf6 (Figures 1C,D). These results raised the possibility that Slamf5 could be acting as a SAP-dependent negative regulator of innate CD8^+^ T cell expansion in the periphery.

The above findings also suggest that the requirement of NKT cells during innate CD8^+^ T cell development and/or expansion is not absolute. To test this, we looked for the presence of innate-like CD8^+^ T cells in *Jα18^−/−^* B6 mice, which, like *Sh2d1a^−/−^* B6 mice, completely lack NKT cells (59). Similar to what we found in *Slamf[1 *+ *5 *+ *6]^−/−^*mice (Figures 1C,D), *Jα18^−/−^* B6 mice did not present significant alterations in the innate CD8^+^ T cell compartment, showing similar percentages of these cells both in the thymus and spleen compared to WT (Figures 1E,F). Intriguingly, although SAP was shown to be required for the selection of innate-like CD8^+^ T cells in the *Itk^−/−^* background (40), we found a significant increase of these cells in the thymus of *Sh2d1a^−/−^* B6 mice (Figures 1E,F). Together, these results show that in the absence of NKT cells, innate-like CD8^+^ T cells can develop relatively normally in the thymus and spleen of B6 mice.

### Slamf5 is a negative regulator of innate CD8^+^ T cell expansion

**Figure 2:**
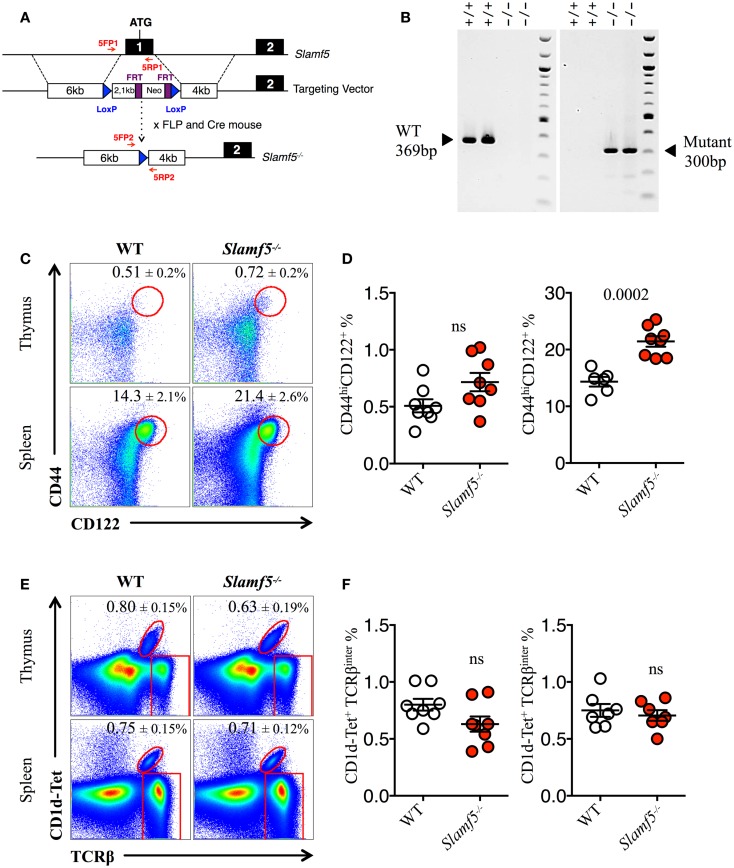
**Slamf5 acts as a negative modulator of innate CD8^+^ T cell**. **(A)** Schematic representation of the Slamf5 conditional targeting strategy. Homologous recombination of the targeting construct with the *Slamf5* genomic locus results in the introduction of an FRT-Neo-FRT cassette along with two LoxP sites surrounding exon 1. Subsequent FLP- and Cre-mediated recombination events lead to the generation of the *Slamf5^−/−^* gene. **(B)** Mouse tail DNA was isolated and screened by PCR using the two sets of primers (5FP1 + 5RP1 and 5FP2 + 5RP2) depicted in **(A)**. Fragments of ~369 and ~300 base pairs are expected for the WT and mutant alleles, respectively. The PCR gel shows a representative result of two WT and two *Slamf5^−/−^* mouse DNA samples. **(C)** Representative FACS plots showing the expression of CD44 and CD122 on CD8SP T cells from thymus (TCRβ^hi^) or spleen (TCRβ^+^) of WT or *Slamf5^−/−^* B6 mice. FACS plots are gated on live singlets lymphocytes TCRβ^hi^ CD8SP (*thymus*) or TCRβ^+^ CD8SP (*spleen*). **(D)** Percentage of CD44^hi^CD122^+^ CD8SP T cells in thymus (*left*) or spleen (*right*) of WT or *Slamf5^−/−^* B6 mice. **(E)** Representative FACS plots showing PBS-57 CD1d-tetramer (CD1d-Tet) reactive NKT cells from thymus or spleen of WT or *Slamf5^−/−^* B6 mice. FACS plots are gated on live singlets lymphocytes. **(F)** Percentage of CD1d-Tet^+^TCRβ^inter^ cells in thymus (*left*) or spleen (*right*) of WT or *Slamf5^−/−^*mice. Numbers inside plots show the mean percentage ± SD of innate-like CD8^+^ T cells **(C)**, or NKT cells **(E)**. Red rectangles in **(E)** depict the lymphocyte gate in which innate CD8^+^ T cells were analyzed. Cumulative graphs show the results of three independent experiments, mean ± SEM. The *p* values were calculated by unpaired *t*-test between the WT group and the *Slamf5^−/−^* group. A value of *p * < 0.05 was considered significant; ns, non-significant.

The enlarged population of CD44^hi^CD122^+^ CD8^+^ T cells found in the spleen of *Slamf[1* + *5 *+ *6]^−/−^*mice but not in the spleen of *Slamf[1* + *6]^−/−^* mice (Figures 1C,D) prompts us to assess whether the Slamf5/SAP axis could be negatively regulating the development of these non-conventional lymphocytes. Therefore, we used the single-knockout *Slamf5^−/−^* mouse to check this hypothesis (Figures 2A,B). Indeed, we found a significant increase in the percentage and absolute counts (*data not shown*) of innate-like CD8^+^ T cells in the spleen of *Slamf5^−/−^* mice compared to WT B6 mice. In the thymus, we observed a similar trend, but this result did not reach statistical significance (Figures 2C,D). However, the NKT cell compartment of *Slamf5^−/−^* mice appeared slightly reduced by the absence of this receptor (Figures 2E,F). Together, these data suggest that the SAP-binding receptor Slamf5 may be acting as a negative regulator of innate CD8^+^ T cell development and/or expansion in B6 mice (Table 2).

### SAP-independent receptor Slamf7 negatively controls the development of innate lymphocytes in B6 mice

**Figure 3:**
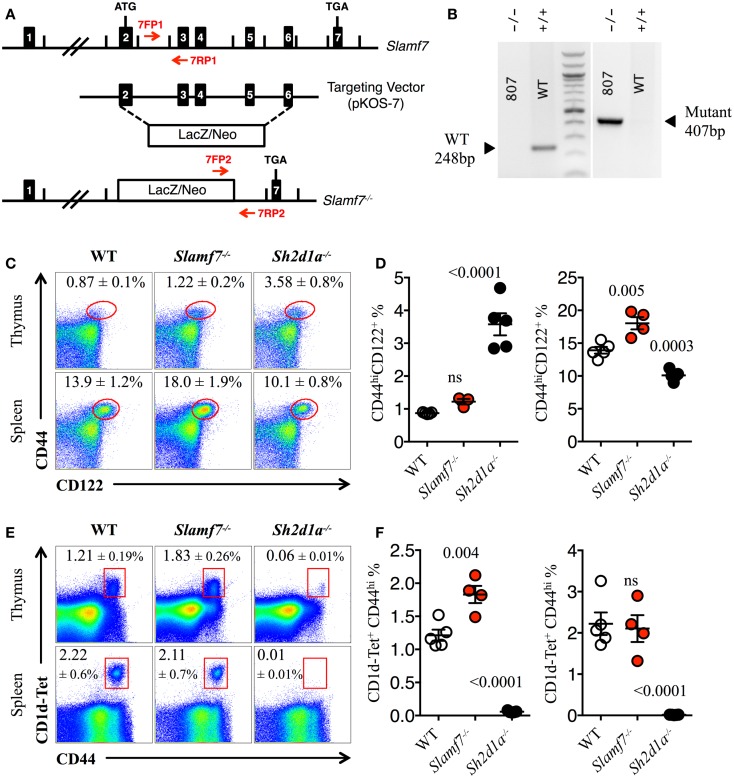
**Expansion of innate CD8^+^ T cells in the absence of Slamf7**. **(A)** Schematic representation of the *Slamf7^−/−^* mice generation. The pKOS-7 targeting vector was designed to replace a genomic fragment containing exon 2–6 with a LacZ/Neo cassette. **(B)** Mouse tail DNA was isolated and screened by PCR using the two sets of primers (7FP1 + 7RP1 and 7FP2 + 7RP2) depicted in **(A)**. Fragments of ~248 and ~407 base pairs are expected for the WT and mutant alleles, respectively. The PCR gel shows a representative result of one WT and one *Slamf7^−/−^* DNA mouse samples. **(C)** Representative FACS plots showing the expression of CD44 and CD122 on CD8SP T cells from thymus or spleen of WT, *Slamf7^−/−^* or *Sh2d1a^−/−^* B6 mice. FACS plots are gated on live singlets lymphocytes TCRβ^hi^ CD8SP (*thymus*) or TCRβ^+^ CD8SP (*spleen*). **(D)** Percentage of CD44^hi^CD122^+^ CD8SP T cells in thymus (*left*) or spleen (*right*) of WT, *Slamf7^−/−^* or *Sh2d1a^−/−^* B6 mice. **(E)** Representative FACS plots showing PBS-57 CD1d-tetramer (CD1d-Tet) reactive NKT cells from thymus or spleen of WT, *Slamf7^−/−^* or *Sh2d1a^−/−^* B6 mice. FACS plots are gated on live singlets lymphocytes. **(F)** Percentage of CD1d-Tet^+^CD44^+^ cells in thymus (*left*) or spleen (*right*) of WT, *Slamf7^−/−^* or *Sh2d1a^−/−^* B6 mice. Numbers inside plots show the mean percentage ± SD of innate-like CD8^+^ T cells **(C)** or NKT cells **(E)**, mean ± SEM. The *p* values were calculated by unpaired *t*-test between the WT group and *Slamf7^−/−^* or *Sh2d1a^−/−^* group. A value of *p * < 0.05 was considered significant; ns, non-significant.

Next, we evaluated whether Slamf7 deficiency (Figures 3A,B) could also alter the innate CD8^+^ and/or NKT cell compartment of B6 mice. We found a significant expansion of CD44^hi^CD122^+^ innate-like CD8^+^ T cells in the spleen but not in the thymus of *Slamf7^−/−^* mice as compared to WT B6 controls (Figures 3C,D). In contrast, and as shown in Figures 1E,F, *Sh2d1a^−/−^* B6 mice displayed a clear expansion of CD44^hi^CD122^+^ innate-like CD8^+^ T cells in the thymus (Figures 3C,D). We also observed a significant increase of CD44^hi^ mature NKT cells in the thymus of *Slamf7^−/−^* mice (Figures 3E,F). As expected, *Sh2d1a^−/−^* B6 mice almost completely lack mature NKT cells (Figures 3E,F). These results suggest that endogenous expression of Slamf7 negatively influences the fate of both innate CD8^+^and NKT cells. Whether this occurs via EAT-2A, a second adaptor that was shown to bind the Slamf7 receptor (23, 24) or via SAP recruitment to the cytoplasmic tail of Slamf7 remains to be determined.

### Expansion of innate-like T cells in *Slamf8^−/−^* BALB/c mice

**Figure 4:**
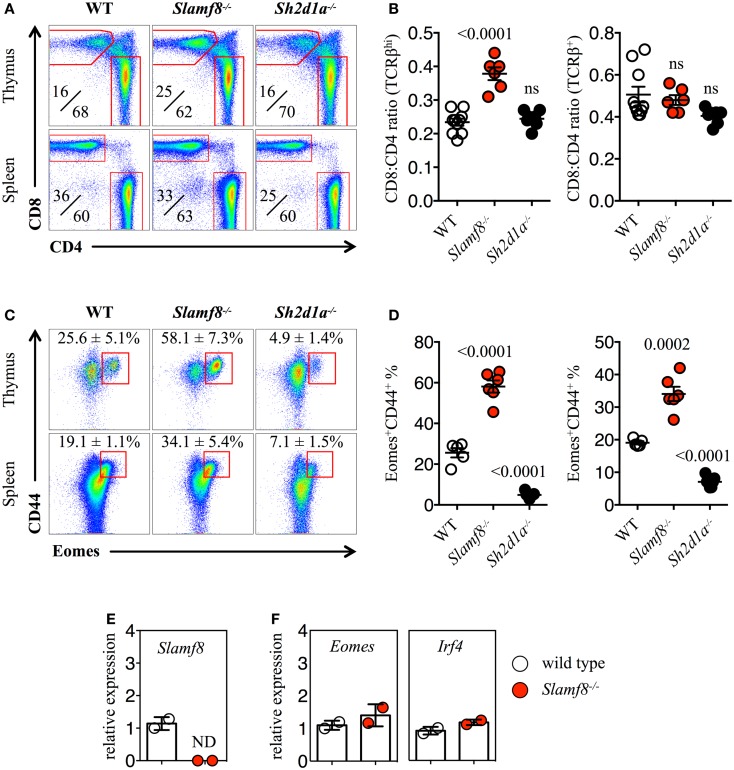
**Lack of *Slamf8* expression triggers an expansion of Eomes^+^ CD8^+^ T cells**. **(A)** Representative FACS plots showing CD4 and CD8 single positive (SP) T cells from thymus or spleen of WT, *Slamf8^−/−^* or *Sh2d1a^−/−^* BALB/c mice. FACS plots are gated on live singlets TCRβ^hi^ (*thymus*) or TCRβ^+^ (*spleen*) lymphocytes. **(B)** CD8:CD4 ratio of TCRβ^hi^ thymocytes (*left*) or TCRβ^+^ splenocytes (*right*) from WT, *Slamf8^−/−^* or *Sh2d1a^−/−^* BALB/c mice. **(C)** Intracellular staining for the transcription factor Eomesodermin (Eomes) in thymus or spleen of WT, *Slamf8^−/−^* or *Sh2d1a^−/−^* BALB/c mice. FACS plots are gated on live singlets lymphocytes TCRβ^hi^ CD8SP (*thymus*) or TCRβ^+^ CD8SP (*spleen*). **(D)** Percentage of Eomes^+^CD44^+^ CD8SP T cells in thymus (*left*) or spleen (*right*) of WT, *Slamf8^−/−^* or *Sh2d1a^−/−^* BALB/c mice. **(E)** The relative mRNA expression of *Slamf8*, or *Eomes* and *Irf4* **(F)** was determined in total thymocytes from WT or *Slamf8^−/−^* BALB/c mice by real-time PCR. Results were normalized to the expression of the housekeeping gene 18S rRNA and expressed relative to WT BALB/c levels (WT = 1); ND, not detected. Numbers inside plots show the mean percentage **(A)** or the mean percentage ± SD **(C)** of the indicated population. Cumulative graphs show the results of four independent experiments, mean ± SEM. The *p* values were calculated by unpaired *t*-test between the WT group and the *Slamf8^−/−^* or *Sh2d1a^−/−^* group. A value of *p * < 0.05 was considered significant; ns, non-significant.

To date, nothing is currently known about the contribution of the SAP-independent receptor Slamf8 during T cell development. Thus, in order to test whether the lack of Slamf8 may influence this process, we systematically identified specific T cell subsets present in the thymus and spleen of *Slamf8^−/−^* BALB/c mice by FACS (Figure 4E; Figure S2A in Supplementary Material). We also included SAP-deficient BALB/c mice (*Sh2d1a^−/−^* BALB/c) in these studies in order to evaluate the role of this SLAMF adaptor in a murine background other than B6. We readily identified an expansion of TCRβ^hi^ CD8SP T cells in the thymus of *Slamf8^−/−^* mice compared to WT BALB/c mice. This alteration appeared to be specific for this organ, as this was not observed in the spleen of *Slamf8^−/−^* mice (expressed as a CD8:CD4 ratio; Figures 4A,B), a phenotype resembling *Slamf3^−/−^* mice on the same background (22) (Table 3).

**Table 3:** **Effects of SAP and SLAMF receptor deficiencies on innate T cell development in the thymus of BALB/c mice**.

BALB/c	*Sh2d1a^−/−^*	*F1^−/−^*	*F3^−/−^*	*F8^−/−^*
Mature NKT	↓ ↓ ↓	=	↑ ↑ ↑	±
Innate CD8	↓ ↓ ↓	↓ ↓	↑ ↑ ↑	↑ ↑ ↑
References	This work	Unpublished observations	(22)	This work

*↓, Reduction; ↑, expansion; ±, marginal/some effect; =, no effect*.

Because of the CD8-biased T cell expansion observed in *Slamf8^−/−^* BALB/c mice, we analyzed the intracellular expression of the CD8-specific T-box transcription factor Eomes. Consistent with the above results, we found a twofold increase in the proportion of Eomes^+^ CD8SP T cells in the thymus of *Slamf8^−/−^* mice (Figures 4C,D). Interestingly, and despite the comparable ratio of CD8:CD4 T cells found in the spleen of *Slamf8^−/−^* mice, Eomes^+^ CD8SP T cells also increased in this organ compared to WT mice (Figures 4C,D). In contrast, *Sh2d1a^−/−^* mice, which displayed no change in CD8:CD4 ratios in thymus or spleen, showed a dramatic decrease in Eomes^+^ CD8^+^ T cells (Figures 4C,D). On the other hand, the relative expression of *Eomes* and *Irf4* (IFN regulatory factor 4) mRNA, whose expression was shown to inversely correlate with that of *Eomes* in recently activated CD8^+^ T cells (3, 60), did not differ between *Slamf8^−/−^* and WT resting thymocytes (Figure 4F). In conclusion, the absence of Slamf8 expression favors the expansion of a CD8SP T cell population expressing Eomes in thymus and spleen. Moreover, although the absence of SAP expression does not alter the CD8:CD4 ratios in thymus or spleen, it dramatically decreases the frequency of Eomes^+^ CD8SP T cells in these organs.

### CD44^hi^CD122^+^ CD8SP T cells in *Slamf8^−/−^* mice express the transcription factor Eomes and rapidly produce IFN-γ upon stimulation

**Figure 5:**
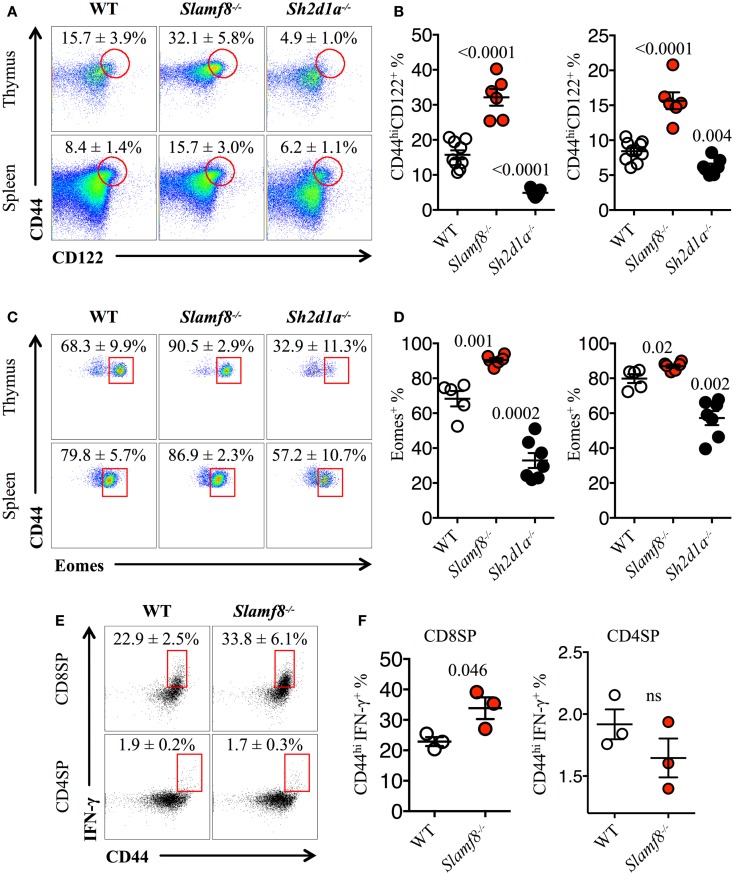
***Slamf8^−/−^* mice have an enlarged CD8^+^ T cell compartment with innate-like features**. **(A)** Representative FACS plots showing the expression of CD44 and CD122 on CD8SP T cells from thymus or spleen of WT, *Slamf8^−/−^* or *Sh2d1a^−/−^* BALB/c mice. FACS plots are gated on live singlets lymphocytes TCRβ^hi^ CD8SP (*thymus*) or TCRβ^+^ CD8SP (*spleen*). **(B)** Percentage of CD44^hi^CD122^+^ CD8SP T cells in thymus (*left*) or spleen (*right*) of WT, *Slamf8^−/−^* or *Sh2d1a^−/−^* BALB/c mice. **(C)** Intracellular staining for the transcription factor Eomesodermin (Eomes) in thymus or spleen of WT, *Slamf8^−/−^* or *Sh2d1a^−/−^* BALB/c mice. FACS plots are gated on live singlets lymphocytes CD44^hi^CD122^+^TCRβ^hi^CD8SP T cells. **(D)** Percentage of Eomes^+^ innate-like CD8^+^ T cells in thymus (*left*) or spleen (*right*) of WT, *Slamf8^−/−^* or *Sh2d1a^−/−^* BALB/c mice. **(E)** Intracellular staining for IFN-γ after *ex vivo* activation with PMA and ionomycin of WT or *Slamf8^−/−^* BALB/c thymocytes. **(F)** Percentage of CD44^hi^IFN-γ^+^ CD8SP (*left*) or CD44^hi^ CD4SP (*right*) of WT or *Slamf8^−/−^* BALB/c thymocytes. Numbers inside plots show the mean percentage ± SD of the indicated population. Cumulative graphs show the results of four independent experiments, mean ± SEM. The *p* values were calculated by unpaired *t*-test between the WT group and the *Slamf8^−/−^* or *Sh2d1a^−/−^* group. A value of *p * < 0.05 was considered significant; ns, non-significant.

Consistent with the enlarged Eomes^+^CD8^+^ T cell compartment in *Slamf8^−/−^* mice, we found a larger proportion of CD44^hi^CD122^+^ innate-like CD8^+^ T cells in these mice (Figures 5A,B), whereas *Sh2d1a^−/−^* mice showed a significant reduction of these cells in the thymus and spleen (Figures 5A,B). Interestingly, within the thymic innate-like CD8^+^ T cell population, *Slamf8^−/−^* mice showed a higher proportion of Eomes^+^ cells compared to WT mice (Figures 5C,D), supporting the idea that the expanded CD8^+^ T cell population documented in Figure 4 corresponds to truly *bona fide* innate CD8^+^ T cells. Consistent with a reduced CD44^hi^CD122^+^ CD8SP T cell population, *Sh2d1a^−/−^* BALB/c mice displayed a dramatic reduction in Eomes^+^ CD8SP T cells in the thymus and, to a lesser extent, in the spleen as well (Figures 5C,D). These seemingly contrasting results, compared to *Sh2d1a^−/−^* B6 mice (Figures 1 and 3; Table 2), indicate that the consequences of a SAP mutation will be greatly influenced by the mouse background. In fact, this phenomenon was previously observed in *Slamf3^−/−^* mice in which profound alterations of the innate CD8^+^T cell compartment were only present on the BALB/c but not on the B6 background (22, 58) (Table 3).

Finally, to confirm that the expanded population of innate-like CD8^+^ T cells found in *Slamf8^−/−^* mice had the ability to produce effector cytokines upon activation, we stimulated total thymocytes *ex vivo* with PMA plus ionomycin. This treatment resulted in a higher percentage of IFN-γ producing *Slamf8^−/−^* CD8SP T cells compared to WT cells (Figures 5E,F). Together, these data demonstrate that the lack of Slamf8 expression results in a preferential expansion of CD8^+^ T cells with an innate-like phenotype. These cells express the transcription factor Eomes and produce IFN-γ upon stimulation, suggesting that SAP-independent signals can also modulate the generation of non-conventional CD8^+^ T cells in mice.

### *Slamf8^−/−^* mice have an enlarged proportion of NKT PLZF^hi^ cells in the thymus

**Figure 6:**
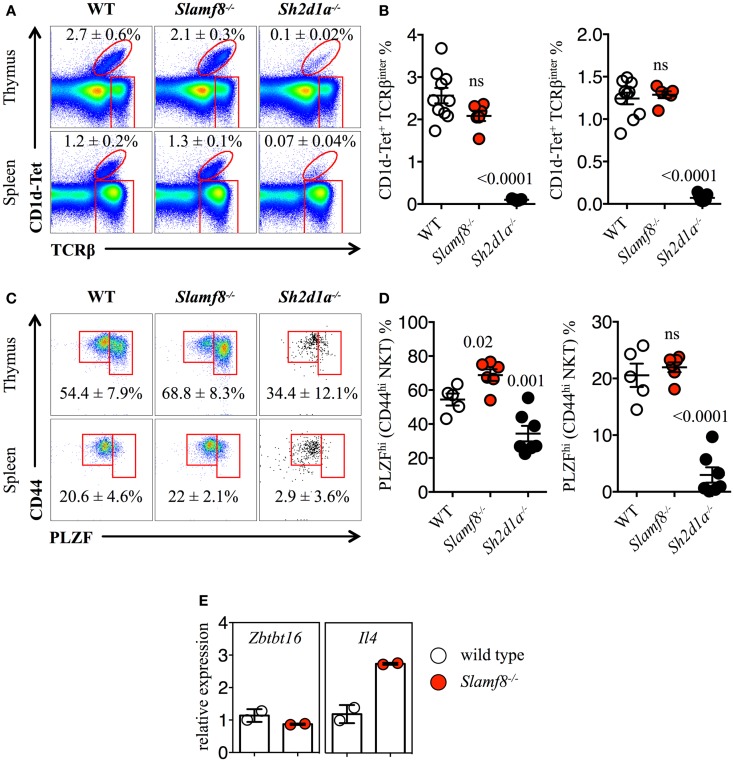
**Increased percentage of PLZF^hi^ NKT cells in *Slamf8^−/−^* mice**. **(A)** Representative FACS plots showing PBS-57 CD1d-tetramer (CD1d-Tet) reactive NKT cells from thymus or spleen of WT, *Slamf8^−/−^* or *Sh2d1a^−/−^* BALB/c mice. FACS plots are gated on live singlets lymphocytes. **(B)** Percentage of CD1d-Tet^+^TCRβ^inter^ cells in thymus (*left*) or CD1d-Tet^+^TCRβ^+^ in spleen (*right*). **(C)** Intracellular staining for the transcription factor promyelocytic leukemia zinc finger (PLZF) in thymus or spleen of WT, *Slamf8^−/−^* or *Sh2d1a^−/−^* BALB/c mice. FACS plots are gated on live singlets CD1d-Tet^+^TCRβ^inter^ cells. **(D)** Percentage of PLZF^hi^ NKT cells in thymus (*left*) or spleen (*right*) of WT, *Slamf8^−/−^* or *Sh2d1a^−/−^* BALB/c mice. **(E)** The relative mRNA expression of *Zbtb16* (encoding PLZF) and *IL-4* (encoding Interleukin-4) was determined in total thymocytes from WT or *Slamf8^−/−^* BALB/c mice by real-time PCR. Results were normalized to the expression of the housekeeping gene 18S rRNA and expressed relative to WT BALB/c levels (WT = 1). Numbers inside plots show the mean percentage ± SD of NKT cells **(A)**, or PLZF^hi^ NKT cells for **(C)**. Red rectangles in **(A)** depict the lymphocyte gate in which innate CD8^+^ T cells were analyzed. Cumulative graphs show the results of three independent experiments, mean ± SEM. The *p* values were calculated by unpaired *t*-test between the WT group and the *Slamf8^−/−^* or *Sh2d1a^−/−^* group. A value of *p * < 0.05 was considered significant; ns, non-significant.

Recent studies have strongly suggested that thymic NKT cells can change the size of the innate CD8^+^ T cell pool via the production of IL-4 (10, 22, 42, 43, 61). Therefore, we evaluated whether we could find an expansion of NKT cells in the thymus and spleen of *Slamf8^−/−^* mice. Unlike *Slamf3^−/−^* BALB/c mice, in which an expansion of NKT cells was reported (22), we did not observe significant differences in the percentage of total NKT cells in the thymus or spleen of *Slamf8^−/−^* mice compared to WT mice (Figures 6A,B). Accordingly, and consistent with an essential role of SAP during NKT cell development on B6 mice (17–19), *Sh2d1a^−/−^* BALB/c mice were almost completely devoid of these cells (Figures 6A,B). These observations unmistakably demonstrate that SAP-derived signals contribute enormously to the NKT cell lineage commitment independently of the mouse genetic background. Moreover, the lack of Slamf8 does not greatly affect the proportions of CD1d-Tet reactive NKT cells.

Just as Eomes controls the effector program of innate CD8^+^ T cells (44), NKT cells in the thymus rely on the expression of PLZF to gain their phenotypical and functional properties (62–65). Although we did not observe significant differences in the percentage of CD44^hi^ NKT cells between WT and *Slamf8^−/−^* mice (Figures 6A,B), the proportion of PLZF^hi^CD1d-Tet reactive cells was slightly higher both in the thymus and spleen of *Slamf8^−/−^* mice, as judged by intracellular PLZF staining (Figures 6C,D). In contrast, we found a significant reduction of the PLZF^hi^ population in the few NKT cells that we could detect in *Sh2d1a^−/−^* BALB/c mice (Figures 6C,D). However, we did not detect differences in *Zbtb16* mRNA expression (encoding PLZF) between WT and *Slamf8^−/−^* resting thymocytes. On the other hand, an approximately threefold increase in *IL-4* mRNA (encoding IL-4) was detected in *Slamf8^−/−^* samples (Figure 6E). Together, these data show that the absence of Slamf8 expression licenses the expansion of both Eomes^+^ CD8SP and PLZF^hi^ NKT cells preferentially in the thymus, and that those NKT cells are *biased* to produce IL-4, which in turn may contribute to the expansion of innate CD8^+^ T cells in *Slamf8^−/−^* mice. This proposes an unprecedented role for the SAP-independent SLAMF receptor Slamf8 in regulating innate T cell development.

## Discussion

Inadequate TCR-related signaling during T cell development, including overly strong or weak MHC/TCR interactions, results in the deletion of up to 90% of developing thymocytes (66). SLAMF receptors and the adaptor SAP have been shown to play an essential modulatory role during selection of non-conventional T cells, including NKT and innate CD8^+^ T cells. These homophilic interactions provide developing NKT cells, and likely innate CD8^+^ T cells, with both positive and negative developmental cues. Interestingly, the SLAMF/SAP pathway does not seem to affect conventional αβ T cell development, suggesting the existence of a specialized mechanism by which innate lymphocytes are preferentially generated in the thymus. These observations could be partially explained by the particular *modus operandi* of the SLAMF receptors, in which, as self-ligands, homotypic cell–cell contacts can be specially favored by homophilic SLAMF–SLAMF interactions. Additionally, as the expression of SLAMF receptors is confined to the hematopoietic compartment, interactions of SLAMF-expressing DP thymocytes with thymic epithelial cells cannot lead to a productive activation of the SLAMF/SAP signaling cascade in those cells. On the other hand, if two interacting DP thymocytes display a similar SLAMF receptor expression pattern, then their homophilic SLAMF–SLAMF interactions will be particularly favored, leading to a successful expansion of these clones.

The generation of double and triple SLAMF-deficient mouse strains allowed us for the first time to study the simultaneous effects of a total abolition of Slamf1, Slamf5, and Slamf6 on NKT and innate CD8^+^ T cell development. Unlike our previous experiments using mixed-bone marrow chimeras (20), these mice provided us with the unique opportunity to avoid any possible unwanted effects derived from the *sterile* inflammation induced by the gamma radiation and/or the possible contribution of radio-resistant cells inherent in these kinds of experiments. Since both *Slamf[1 *+ *6]^−/−^* and *Slamf[1 *+ *5 *+ *6]^−/−^*mice failed to recapitulate the total NKT cell developmental arrest found in *Sh2d1a^−/−^*mice (20) (Figure 1), we conclude that there must be yet another SLAMF receptor that is able to recruit SAP in the absence of Slamf1, Slamf5, and Slamf6. As we could not detect Slamf4 expression in DP thymocytes, we ruled out both Slamf2 and Slamf4 from the list of possible candidates. Since *Slamf3^−/−^* B6 mice were reported not to have a decrease in NKT cell number (58), we can also rule out this receptor. We then tested *Slamf5^−/−^* and *Slamf7^−/−^* B6 mice. Surprisingly, instead of a decrease in NKT and/or innate CD8^+^ T cells, we found a significant increase of both these cell populations in these mice (Figures 2 and 3), suggesting that these receptors act as negative regulators during innate T cell development. However, as it was shown for Slamf6 expression, in which this receptor was able to provide both positive and negative signals to NKT cell development in a SAP-dependent manner (53), it is still possible that in the presence of other SLAMF receptors, Slamf5 and Slamf7 play opposite roles to the signals coming from Slamf1 and Slamf6. Further experiments will hopefully shed light on this rather intricate but important relationship.

Interestingly, the reduction of NKT cells in *Slamf[1 *+ *6]^−/−^* and *Slamf[1 *+ *5 *+ *6]^−/−^*mice, or the complete absence of these cells in *Sh2d1a^−/−^*B6 mice, did not seem to impair the generation of innate-like CD8^+^ T cells (Figure 1; Table 2). As these results contradicted published evidence showing that NKT cells support the expansion of innate CD8^+^ T cells via IL-4 production (10), and that SAP expression is required for thymic selection of innate-like CD8^+^ T cells in *Itk^−/−^* mice (40), we sought to test if the absence of NKT cells in an otherwise *normal* SAP context would also affect innate CD8^+^ T cell development. Hence, we analyzed the presence of innate-like CD8^+^ T cells in *Jα18^−/−^* mice (Figures 1E,F). Similar to our previous observations in *Sh2d1a^−/−^* B6 mice, we did not detect a reduction of CD44^hi^CD122^+^ cells in this NKT-deficient strain (Figures 1E,F), suggesting that in the absence of NKT cells other cells can provide the factors needed to accomplish this task.

Since all murine SLAMF receptors that have one or more cytoplasmic ITSM, with the exception of Slamf2, Slamf8, and Slamf9, can theoretically recruit SAP and/or EAT-2A, a redundancy in their function during innate cell development can be expected. Supporting this idea, none of the single SLAMF-deficient mice generated on the B6 background have shown substantial alterations in their innate T cell compartments (Table 2). Remarkably, however, *Slamf3^−/−^* (22) and now *Slamf8^−/−^* mice (*this work*), both on the BALB/c background, show massive expansions of innate-like CD8^+^ T cells in the thymus (Table 3), albeit to a far lesser extent than the *Itk^−/−^* or *Rlk^−/−^Itk^−/−^* B6 mice. These results strongly suggest that the mouse background can somehow act as a *pressure selector* of innate T cell development.

The recent identification of a specialized NKT cell subset present in BALB/c mice, but practically absent in B6 mice, capable of constantly producing IL-4 (termed NKT2), allowed Lee et al. to propose a model in which NKT2 cells are readily available in the thymus to secrete IL-4, resulting in intrinsically greater frequencies of innate CD8^+^ T cells (43) on this mouse background. Notably, these cells express the highest levels of PLZF among all mature NKT cells. Consequently, an involvement of NKT2 cells in supporting the expansion of innate-like CD8^+^ T cells in *Slamf8^−/−^* mice can be anticipated, as we find an enlarged PLZF^hi^ NKT population that correlates with a greater proportion of innate CD8^+^ T cells in these mice (Figures 5 and 6). However, the underlying mechanism by which the absence of Slamf8 favors the expansion of PLZF^hi^ NKT cells remains undetermined. Since *Slamf8* mRNA is expressed by DC and fibroblasts in the thymus, but not DP thymocytes (Table 1), we speculate that these cells may be providing some sort of signal to developing NKT and/or innate CD8^+^ T cells which is affected by the absence of Slamf8. In this context, IL-15 transpresentation has been shown to mediate survival of mature thymic NKT and innate CD8αα^+^ intestinal intraepithelial T cells (67–70). Thus, Slamf8 may be regulating the production and/or transpresentation of this cytokine.

By investigating the impact of two previously uncharacterized SLAMF receptors (Slamf7 and Slamf8) as well as the combined impact of multiple SLAMF receptor deletions (*Slamf[1 *+ *6]^−/−^* and *Slamf[1 *+ *5 *+ *6]^−/−^*mice), we are able to present new evidence that shall complement previous knowledge concerning the individual contributions of SLAMF receptors to NKT and innate CD8^+^ T cell biology. Importantly, the outcomes of our studies show the existence of positive and negative signals derived from different SLAMF receptors (Tables 2 and 3), which together orchestrate innate T cell development. The cellular and molecular mechanisms behind these observations certainly deserve more attention in the future, since these pathways could potentially be targeted in the aid of expanding innate T cell populations in situations where SAP is absent, such as in patients with XLP.

## Conflict of Interest Statement

The authors declare that the research was conducted in the absence of any commercial or financial relationships that could be construed as a potential conflict of interest.

## Supplementary Material

The Supplementary Material for this article can be found online at http://www.frontiersin.org/Journal/10.3389/fimmu.2014.00186/abstract

Figure S1 **General gating strategy for the identification of innate (B6) T cells, and SLAMF receptor expression by FACS**. **(A)** Genomic organization of the nine SLAMF genes, and the two adaptors *Sh2d1b1* and *Sh2d1b2* (encoding EAT-2A and ERT, respectively) on the murine chromosome 1H3. Blue filled boxes with a red frame represent SLAMF receptors containing one or more ITSM. Black empty boxes represent SLAMF receptors that lack ITSM. Red and orange boxes represent the *Sh2d1b1* and *Sh2d1b2* genes, respectively. This diagram also illustrates the *Slamf[1 *+ *6]^−/−^* and *Slamf[1 *+ *5 *+ *6]^−/−^*deletions used in this manuscript. **(B)** FACS plots showing the main gating strategy used on the analysis of NKT and innate CD8^+^ T cells in B6 mice. Upper row from left to right: live cells (DAPI negative), singlets, lymphocytes, and total thymocytes depicting the NKT (CD1d-Tet^+^TCRβ^inter^), the TCRβ^hi^ mature, and TCRβ^low^ immature thymocytes gates. Middle row from left to right: CD4^+^CD8^+^ DP TCRβ^low^ immature thymocytes, mature (CD44^+^NK1.1^+^) NKT cells, TCRβ^hi^ mature T cells [depicting CD8 and CD4 single positive (SP) mature thymocytes], and CD8SP T cells depicting the innate CD8^+^ T cell population in the thymus. Bottom row from left to right, mature (CD44^+^NK1.1^+^) NKT cells, TCRβ^+^ T cells [depicting CD8 and CD4 single positive (SP) splenocytes], and CD8SP T cells depicting the innate CD8^+^ T cell population in the spleen. **(C)** Representative FACS staining of innate CD8^+^ T cells (CD44^hi^CD122^+^TCRβ^+^) showing the corresponding fluorescence minus one (FMO) negative control for the CD122 gate in thymus and spleen from WT cells. **(D)** Representative FACS histograms showing the expression of Slamf1, Slamf2, Slamf3, Slamf4, Slamf5, and Slamf6 on the surface of CD4^+^CD8^+^ DP TCRβ^low^ thymocytes (upper row, *green*), CD1d-tetramer (CD1d-Tet) reactive CD44^hi^ NKT cells (*red*, NKT), or on CD44^hi^CD122^+^TCRβ^hi^ (thymus, *middle row*), or TCRβ^+^ (spleen, *bottom row*) CD8SP T cells (*purple*, innate-like CD8) of 10-week-old B6 mice. Gray histograms represent the FMO negative control for the Slamf staining.

Figure S2 **General gating strategy for the identification of innate (BALB/c) T cells by FACS**. **(A)** FACS plots showing the main gating strategy used on the analysis of NKT and innate CD8^+^ T cells in BALB/c mice. Upper row from left to right: live cells (DAPI negative), singlets, lymphocytes, and total thymocytes depicting the NKT (CD1d-Tet^+^TCRβ^inter^), and the TCRβ^hi^ mature lymphocyte gates. Bottom row from left to right: total live thymocytes depicting the CD8 single positive (SP), the CD4SP, and the CD4^+^CD8^+^ DP TCRβ^low^ immature thymocytes. **(B)** Representative FACS staining of innate CD8^+^ T cells (CD44^hi^CD122^+^TCRβ^+^) showing the corresponding FMO (fluorescence minus one) negative control for the CD122 gate (*left*), or Eomesodermin (Eomes, *right*) in thymus and spleen from WT cells.
