# Supplementary material for: SAP-Dependent and -Independent Regulation of Innate T Cell Development Involving SLAMF Receptors
Source: Front Immunol. 2014 Apr 23;5:186. doi: 10.3389/fimmu.2014.00186 (PMC4005954; doi:10.3389/fimmu.2014.00186)

Supplementary Figure S2

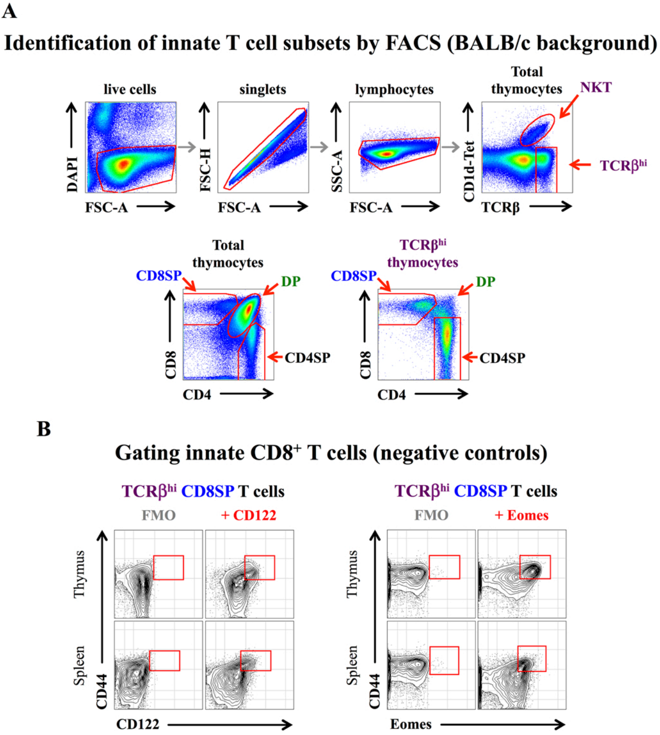

Supplement: Figure S1 — General gating strategy for the identification of innate (B6) T cells, and SLAMF receptor expression by FACS. (A) Genomic organization of the nine SLAMF genes, and the two adaptors Sh2d1b1 and Sh2d1b2 (encoding EAT-2A and ERT, respectively) on the murine chromosome 1H3. Blue filled boxes with a red frame represent SLAMF receptors containing one or more ITSM. Black empty boxes represent SLAMF receptors that lack ITSM. Red and orange boxes represent the Sh2d1b1 and Sh2d1b2 genes, respectively. This diagram also illustrates the Slamf[1 + 6]−/− and Slamf[1 + 5 + 6]−/−deletions used in this manuscript. (B) FACS plots showing the main gating strategy used on the analysis of NKT and innate CD8+ T cells in B6 mice. Upper row from left to right: live cells (DAPI negative), singlets, lymphocytes, and total thymocytes depicting the NKT (CD1d-Tet+TCRβinter), the TCRβhi mature, and TCRβlow immature thymocytes gates. Middle row from left to right: CD4+CD8+ DP TCRβlow immature thymocytes, mature (CD44+NK1.1+) NKT cells, TCRβhi mature T cells [depicting CD8 and CD4 single positive (SP) mature thymocytes], and CD8SP T cells depicting the innate CD8+ T cell population in the thymus. Bottom row from left to right, mature (CD44+NK1.1+) NKT cells, TCRβ+ T cells [depicting CD8 and CD4 single positive (SP) splenocytes], and CD8SP T cells depicting the innate CD8+ T cell population in the spleen. (C) Representative FACS staining of innate CD8+ T cells (CD44hiCD122+TCRβ+) showing the corresponding fluorescence minus one (FMO) negative control for the CD122 gate in thymus and spleen from WT cells. (D) Representative FACS histograms showing the expression of Slamf1, Slamf2, Slamf3, Slamf4, Slamf5, and Slamf6 on the surface of CD4+CD8+ DP TCRβlow thymocytes (upper row, green), CD1d-tetramer (CD1d-Tet) reactive CD44hi NKT cells (red, NKT), or on CD44hiCD122+TCRβhi (thymus, middle row), or TCRβ+ (spleen, bottom row) CD8SP T cells (purple, innate-like CD8) of 10-week-old B6 mice. Gray histograms [file Presentation_1.ZIP › Figure S2.PDF]
